# Assessment of the use of sodium alginate for soil improvement in coastal applications

**DOI:** 10.1038/s41598-025-22427-y

**Published:** 2025-11-05

**Authors:** Gloria M. Castro, Desta Tewelde, Enrico Tubaldi

**Affiliations:** 1 Department of Civil Engineering, University of Birmingham, Birmingham, UK; 2 Department of Civil and Environmental Engineering, University of Strathclyde, Glasgow, UK

**Keywords:** Coastal protection, Sodium alginate, Soil improvement, Bio-mediated, Sustainable infrastructure, Engineering, Environmental sciences, Materials science

## Abstract

Rising sea levels and intensifying coastal erosion necessitate sustainable soil improvement methods to protect vulnerable coastal zones. Previous studies have demonstrated that crosslinked sodium alginate can enhance soil strength. Nevertheless, for coastal applications, it is essential to characterise the effect of increasing alginate concentrations to identify an optimal dosage and to evaluate the stability of the calcium crosslink when exposed to seawater wet-dry cycles. This study explores the potential of sodium alginate as an environmentally friendly technique for improving the stability and performance of granular soils in coastal regions, with the overarching goal of assessing its durability under repeated seawater wet–dry cycles. The experimental work focused on quartzitic, poorly graded sands, beginning with comparisons between different sodium alginates, varying sodium alginate concentrations (1.4–10%) and two mixing methodologies (dry and wet) for treatment. This was followed by an assessment of the effect of seawater wet-dry cycles on the integrity and mechanical behaviour of treated specimens. Unconfined compressive strength (UCS) tests demonstrated a linear increase in strength up to 2.3% alginate concentration, with an optimal UCS achieved at 4.6%. Higher alginate contents led to specimen deformation and reduced strength. Durability assessments involving up to 28 wet-dry cycles with artificial seawater revealed that although calcium alginate membranes maintained chemical stability, specimens exhibited 26–37% reductions in UCS, primarily due to membrane tearing from repeated expansion–contraction and crystallisation pressures from precipitated salts. SEM-EDS analyses confirmed structural damage and the accumulation of sodium chloride, calcium chloride, and calcium sulphate crystals. The results suggest that sodium alginate treatment can effectively improve clean sand strength but supplementary measures are necessary to reduce the permeability of the treated specimens, enhancing their durability under harsh coastal conditions.

**Supplementary Information:**

The online version contains supplementary material available at 10.1038/s41598-025-22427-y.

## Introduction

Global sea level rise has increased from an average rate of 1.3 to 3.7 mm/yr between 1901 and 2018, exacerbating coastal erosion and flood risk worldwide^1^. This poses severe threats to delicate ecosystems, communities and infrastructure near coastlines^2,3^. Coastal storms are also intensifying, with increasing frequency and severity of extreme weather events attributed to climate change.

Current coastal protection strategies often rely on concrete barriers and plastic geosynthetics, which result in damage to natural ecosystems, enhanced microplastic contamination and increased CO_2_ emissions^4–6^. Promising environmentally friendly alternatives include the use of vegetation and soil improvement via bio-mediated processes, such as microbially induced carbonate precipitation (MICP) and biopolymers^7–13^. In particular, sodium alginate (SA), a biopolymer typically derived from brown algae, has displayed promising results as a soil improvement technique^14–18^. SA is a natural polysaccharide most commonly obtained from brown seaweed after a process of drying, milling, acidification, alkali extraction and reprecipitation^19^. The obtained sodium alginate powder is soluble in water, forming a viscous fluid (left-hand side of Eq. 1). Upon contact with dissolved calcium, sodium ions in the alginate chains are exchanged for calcium ions, forming a gel network by ionic cross-links with the carboxylic groups^20^. The resultant calcium alginate is non-soluble in alkaline water and has a membrane-like structure (right-hand side of Eq. 1).1

Previous studies highlight the reversibility of calcium alginate back to sodium alginate if exposed to high sodium chloride concentrations (red arrow in Eq. 1;^21–24^). Efforts to increase the stability of calcium alginate membranes include using high concentrations of sodium alginate (60 g/L^25^), additions of egg proteins^26^, combinations with MICP-capable bacteria^27^, and supplementing graphene oxide fibers^28^.

Two approaches have been used in the literature to improve soil behaviour using powdered sodium alginate:

1. As a viscous binder. Previous studies have explored different application methods (e.g., spraying, submerging, mixing), concentrations (0.25 to 5%), and curing temperatures and times (18–100 °C; 0 to 28 days), achieving a maximum compressive strength of 1.6 MPa^14,15,17,18,29,30^. This methodology can be problematic near water bodies because sodium alginate is soluble in water; and exposure to it will result in loss of strength.
2. As a solid membrane (crosslinking). Upon exposure to dissolved calcium, sodium alginate transforms into calcium alginate, resulting in a solid, insoluble membrane-like structure. A previous study found that alginate concentration relates linearly to increased unconfined compressive strength (UCS) and reduced hydraulic conductivity^16^. The same study showed that three days is the optimum reaction time for sodium alginate samples exposed to calcium chloride and that maximum strength is achieved when cured at 50 °C. However, durability was limited, as after 12 wet-dry cycles with fresh water, specimens displayed a linear decline in compressive strength. The low sodium alginate concentrations used in this study (up to 0.4%), may have resulted in limited stability of the reinforcing membrane. Similarly, Soldo et al. (2020)^8^ emphasized the importance of allowing samples to dry completely, achieving higher UCS values, with a maximum of 2 MPa at 2% alginate content.

An innovative alternative in this line is to combine alginate with acidifying bacteria to obtain the calcium from calcareous sand particles, yet this methodology could weaken the original soil fabric^31^.

Previous studies have demonstrated that crosslinked sodium alginate has potential for soil improvement. However, for coastal applications, several research gaps must be addressed, including the need to characterise the maximum achievable strength to determine the optimal alginate concentration and to evaluate the stability of the calcium crosslink when subjected to seawater wet–dry cycles.

This study addresses these gaps by systematically investigating the use of sodium alginate as a soil improvement agent for coastal environments, with special consideration of the enhanced degradation driven by wet-dry cycles of seawater. Experimental work has focused on quartzitic, poorly graded sands, beginning with comparisons between different types of sodium alginate, varying concentrations, and two treatment methodologies (dry and wet mixing). This was followed by an assessment of the effects of seawater wet–dry cycles on the integrity, chemical compositon, and mechanical behaviour of the treated specimens. The study results provide useful insights into the optimal formulation and application method of sodium alginate for enhancing soil strength and durability in coastal settings, while contributing to the development of more sustainable ground improvement strategies.

The following section illustrates the methodology followed for specimen preparation, and chemical, mechanical and micro-scale characterisations. Section 3 illustrates the results, Sect. 4 presents analysis and discussion, followed by a Conclusion section.

## Methodology

### Materials

#### Sodium alginate

In this study, sodium alginate was sourced from cast stems of Laminaria hyperborea, a species of brown algae commonly found along North Atlantic coastlines. This specific source is known for yielding high-quality alginate with favourable gelling properties, making it particularly suitable for engineering applications. Three variations of sodium alginate were procured in powdered form from Marine Biopolymers, Scotland: (1) Standard Sodium Alginate (SA), (2) Ultra Low Viscosity Sodium Alginate (ULVSA), and (3) Sodium Alginate with higher residual calcium (SAC). All variations had 12% water content.

#### Sand

This study used quarzitic, poorly graded sand with a mean particle diameter *D*_*50*_ = 0.21 mm, sourced from Mineral Marketing Ltd. The material was selected to minimise variability in boundary conditions, providing a chemically inert medium with minimal ionic interaction and uniform packing. The sand was washed with tap water to remove contaminants and dried at 105 °C before sample preparation.

#### Artificial seawater

Tidal action and evaporation can cause slight daily variations in the chemical composition of naturally sourced seawater^32^. To maintain controlled chemical boundary conditions in this study, all experiments used laboratory-prepared seawater. An artificial seawater solution was prepared following typical seawater major ions concentrations per kilogram of solution^33^: 26.52 g sodium chloride (NaCl), 2.45 g magnesium chloride (MgCl_2_), 3.31 g magnesium sulfate (MgSO_4_), 1.14 g calcium chloride (CaCl_2_), and 0.73 g potassium chloride (KCl). Salts were added to a borosilicate glass bottle placed over a scale (tared), then tap water was added gently until the scale registered 1 kg. The container was sealed and mixed thoroughly.

### Sample preparation

**Fig. 1:**
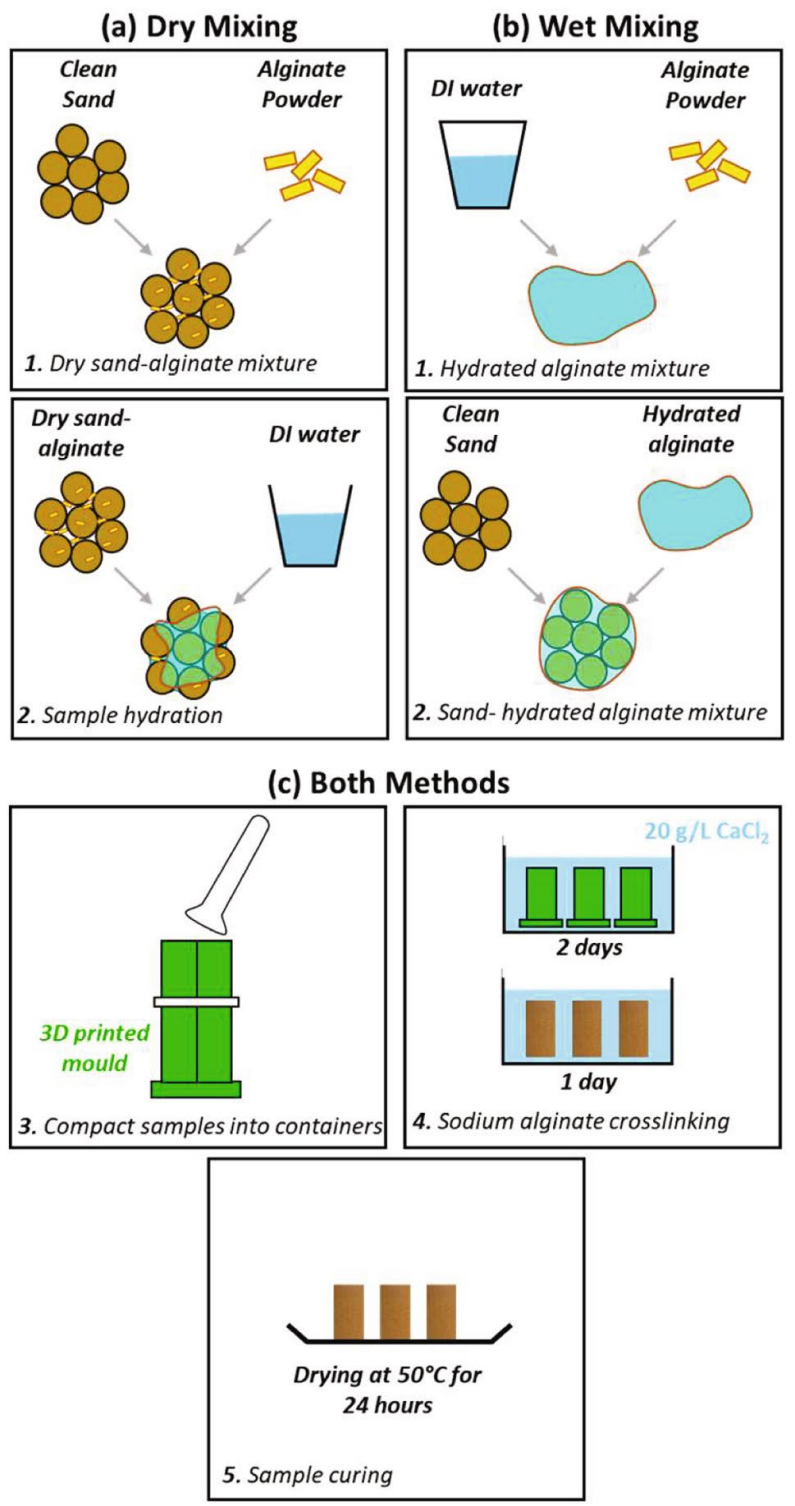
Sample preparation methodologies. (**a**) dry mixing procedures; (**b**) wet mixing processes; and **c** steps performed in both sample preparation procedures.

General procedures are described first, followed by details on the number of samples and sodium alginate concentrations (also provided in Table 1). Sample preparation comprised mixing, compaction, crosslinking and curing (see pictograms in Fig. 1 and photographic images in S1).

**Table 1:** Description of samples prepared in this study.

Alginate concentration [%]	1.4	2.3		4.6	8	10
Mixing method	Wet	Dry	Wet	Dry	Dry	Dry
Alginate moist weight [g]	1.36	2.27		4.52	7.86	9.82
Number of samples	14*	12	12	12	3	3

*12 with SAC, 1 with SA and 1 with ULVSA.

#### Mixing

The use of sodium alginate powder allowed investigation of the effect of hydration prior to mixing with the sand (wet mixing) versus hydration after mixing (dry mixing). Previous studies have highlighted the suitability of each method for deep and shallow biopolymer-based stabilization respectively^34^. Therefore, both methodologies were assessed using identical quantities of materials. Specific procedures follow.

##### Dry mixing

**Fig. 2:**
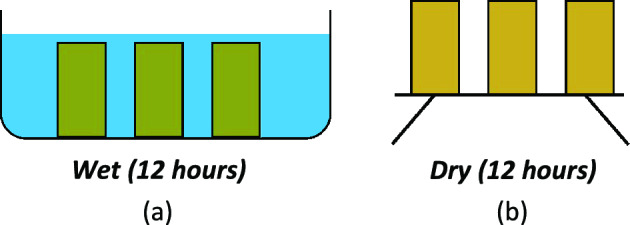
Wet-dry cycles illustration. (**a**) wet event involved submerging the specimens in artificial seawater for 12 h; followed by (**b**) allowing the specimens to drain and dry at room temperature during 12 h in a drying rack.

1. Dry sand (86.45 g) was mixed with alginate powder (Fig. 1a-1).
2. 1PV (20 ml) of DI water was added and mixed thoroughly (Fig. 1a-2).

This method facilitates the mixing process, specifically at high alginate concentrations, where hydrated alginate powder is highly viscous.

##### Wet mixing

1. Alginate powder was hydrated with 1PV of DI water and mix thoroughly (Fig. 1b-1).
2. Then, hydrated alginate solution was mixed with dry sand particles (Fig. 1b-2).

In this method, we ensured that all the powder particles were hydrated in the mixture.

#### Compaction

**Fig. 3:**
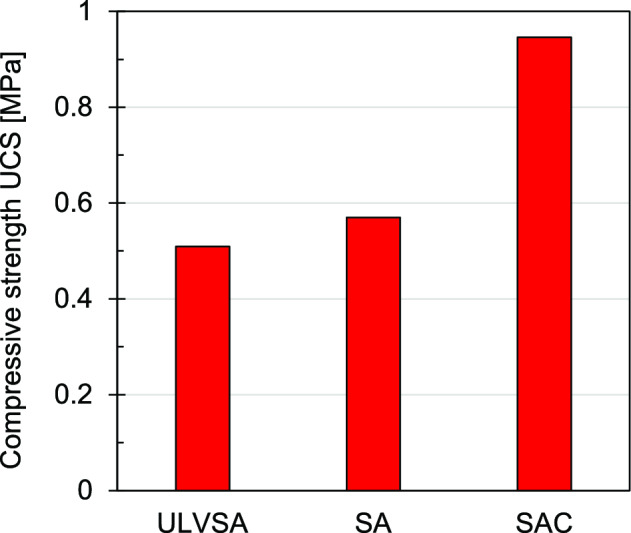
Compressive strength UCS of different sodium alginate powders: SA *Standard Sodium Alginate*, ULVSA Ultra Low Viscosity Sodium Alginate, and SAC Sodium Alginate with higher residual calcium.

Specimens were packed into 3D-printed detachable cylinders (64 mm height, 32 mm diameter) at a void ratio *e* = 0.6 (Fig. 1c-3).

#### Crosslinking

**Fig. 4:**
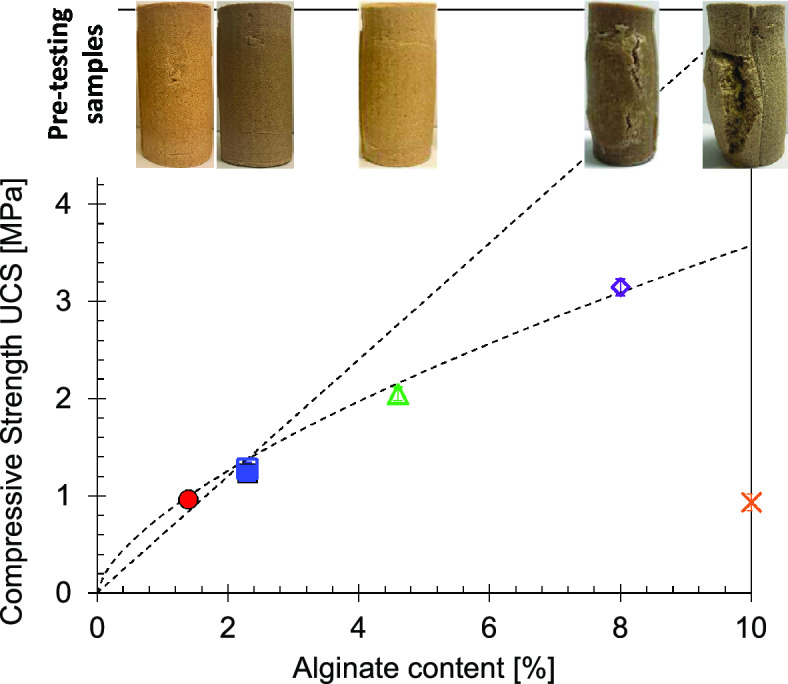
Mixing strategies and material concentrations. Open symbols represent dry mixed, and closed symbols show wet mixed strategies. Data points represent the mean of 3 replicates samples with error bars representing minimum and maximum values. Pictures at the top show specimens after sample preparation (before mechanical testing).

Specimens were submerged in 20 g/L CaCl_2_ solution (216 ml per specimen), ensuring full immersion (Fig. 1c-4). After two days, the moulds were removed and the exposed specimens were left submerged in the CaCl_2_ solution for an additional day to complete crosslinking. Samples were then rinsed with tap water to remove excess salts.

#### Curing

**Fig. 5:**
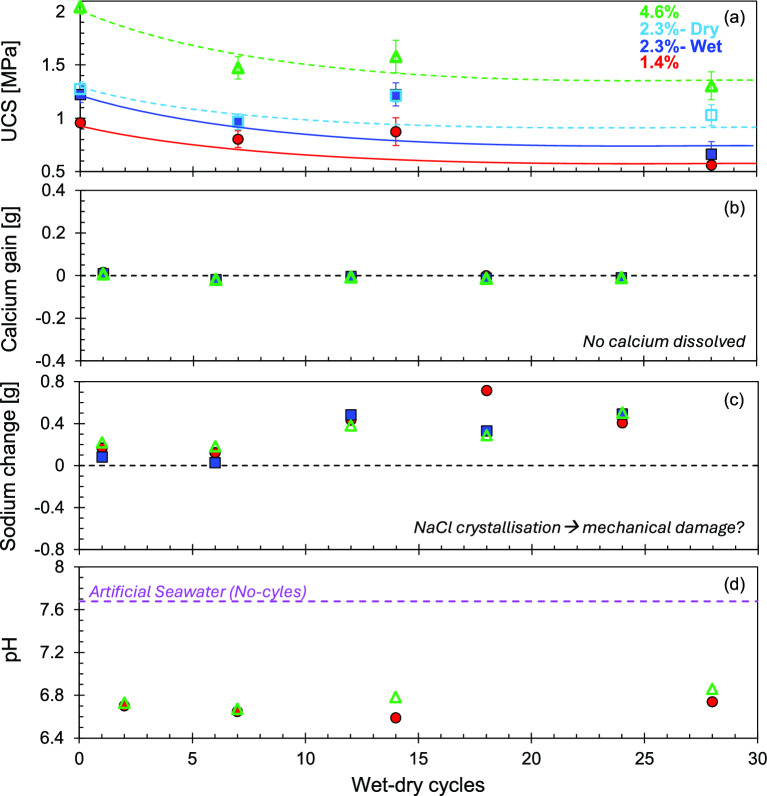
Characterisation after wetting and drying cycles. Open symbols represent dry mixed, and closed symbols show wet mixed strategies. (**a**) unconfined compressive strength UCS results after 0, 7, 14, and 28 cycles. Data points represent the mean of three replicates with error bars representing minimum and maximum values; (**b**) calcium gain; **c** sodium change; **d** pH of artificial seawater before and after cycles.

Samples were dried at 50 °C for 24 h, based on the optimal curing conditions identified by Wen et al. (2019) (^16^, Fig. 1c-5).

#### Description of samples prepared and sodium alginate concentrations

Moist alginate powder content was varied to achieve target concentrations (alginate concentration [%] = dry alginate/dry sand × 100%, Table 1). The minimum concentration (1.4%) was selected based on previous studies suggestions to enhance the stability of crosslinked sodium alginate when exposed to dissolved sodium ions (e.g. 60 g/L^25^). The upper limit (10%) was determined upon observing excessive deformation in prepared specimens.

The study included a total of 56 specimens (Table 1): three for selecting the optimal alginate type at 1.4% (SAC, SA, ULVSA); 24 to assess the effect of dry and wet mixing (all prepared with 2.3% SAC for both methodologies); 18 to evaluate the influence of alginate concentration up to 10%; and 48 to study the effects of wet-dry cycles. Specimens with alginate concentrations ≥ 4.6% were prepared using dry mixing due to the high viscosity of hydrated alginate. Note that 2.3% was selected for comparing wet and dry mixing based on experimental determination of the maximum amount of alginate powder that could be fully dissolved in a fixed volume of deionised water.

### Wet-dry cycles

Each wetting event involved fully submerging specimens in artificial seawater for 12 h (216 ml/specimen). Specimens were subsequently placed in a drying rack for 12 h, where they were allowed to drain and dry at room temperature (Fig. 2). Following each cycle, a sample of the seawater was retained and the remainder discarded, ensuring that each subsequent cycle used freshly prepared artificial seawater with known chemical composition. This procedure was designed to represent wetting events characteristic of coastal environments. Samples subjected to cycling were dried at 50 °C for 24 h to ensure consistent preparation procedures with the uncycled samples prior to UCS testing.

Wet-dry cycles were conducted on groups of three specimens, with duplicate sets, to evaluate unconfined compressive strength (UCS) after 7, 14, and 28 cycles at alginate concentrations of 1.4, 2.3 and 4.6% (Table 1).

### Fluid characterisation

The chemical composition of the artificial seawater after cycling was analysed using Inductively Coupled Plasma Optical Emission Spectroscopy (ICP-OES) to determine dissolved calcium and sodium concentrations following cycles 1, 6, 12, 18, and 24. The pH of the artificial seawater was measured with a pH-sensitive electrode.

### Mechanical characterisation

The unconfined compressive strength (UCS) of all treated specimens was determined in accordance with ASTM D2166/D2166M-16 (2016)^35^, employing a Tinius Olsen Universal Testing machine at a constant loading rate of 0.33 mm/min.

### Micro-scale characterisation

Micro-scale analysis of 4.6% alginate specimens subjected to 7, 14 and 28 wet-dry cycles used a Hitachi S-3700 scanning electron microscope (SEM) equipped with an 80 mm X-Max energy-dispersive spectroscopy (EDS) detector (Oxford Instruments). Prior to SEM–EDS analyses, specimens were coated with a ~ 15 nm gold layer.

## Results

### Sodium alginate type

Figure 3 presents the UCS of specimens prepared at equal concentration (1.4%) using Ultra Low Viscosity Sodium Alginate (ULVSA), Standard Sodium Alginate (SA) and Sodium Alginate with high residual calcium (SAC). SAC displayed significantly higher strength compared to ULVSA and SA. This type of sodium alginate has also shown better stability, and lower cost of production (pers. comm. by Marine Biopolymers). Consequently, SAC was selected for subsequent experimental investigations.

### Alginate content

Compressive strength exhibited a linear increase with alginate concentration up to 2.3% (Fig. 4). Each data point represents the mean of three specimens, with error bars showing the minimum and maximum values. The limited variability highlights the uniformity achieved within and across samples based on our methodology. Specimens prepared at higher alginate concentrations displayed pronounced deformation during crosslinking, with substantial UCS reduction observed at 10% content (see pictures in the upper line of Fig. 4). Additionally, specimens prepared with 2.3% alginate using with both dry and wet mixing displayed comparable compressive strengths, suggesting that mixing technique had limited influence at lower alginate contents.

It is noteworthy that significantly higher values of the UCS were achieved in this compared with previous works, due to the use of higher sodium alginate concentrations (3.1 MPa with 8% SA in this study vs. a maximum of 2 MPa reported by^8^). This could be attributed to the type of alginate employed (gelling properties of SA have been revealed to be strongly correlated with ratio of D-mannuronate to L-guluronate), and the higher content of alginate and of calcium in the solution. Nevertheless, the higher strengths at elevated concentrations were offset by enhanced specimen deformation.

### Effect of seawater wet-dry cycles on alginate-treated soils

#### Mechanical performance

Figure 5a shows the measured UCS after 0, 7, 14, and 28 wet-dry cycles for specimens treated with different alginate concentrations. Each datapoint represents the average of three specimens, with error bars indicating maximum and minimum values. Specimens treated with alginate displayed a 26–37% reduction in UCS after 28 wet-dry cycles, independent of alginate concentration. All groups exhibited a pronounced decline in strength during the initial six cycles, with subsequent cycles inducing minimal additional deterioration.

#### Artificial seawater composition after cycle

Dissolved calcium and sodium concentrations in artificial seawater were characterised after cycles 1, 6, 12, 18, and 24. pH was measured for fresh seawater and after cycles 2, 7, 14 and 28.

##### Calcium

There was no detectable change in calcium concentration from the original artificial seawater composition for any alginate concentration specimens (4.6, 2.3 and 1.4%, Fig. 5b). These results provide evidence of the chemical stability of calcium alginate membranes, even after prolonged exposure to sodium rich environments.

##### Sodium

Figure 5c displays a consistent increase in sodium of about 0.4 g in seawater samples collected from the 12^th^ cycle. Results suggest that sodium gradually accumulated in the specimens before leaching into the seawater during subsequent wet-dry events. This accumulation is likely a consequence of water evaporation during the drying phase. After specimens are removed from the wetting event, they are allowed to dry by evaporation and drainage (similar to a coastal asset exposed to tidal variation). During this time, water held within the pore spaces evaporates, leaving behind the salts originally dissolved in the brine.

##### pH

The artificial seawater used for the cycles had a pH = 7.7 (Fig. 5d), which falls within the typical range for seawater (pH = 7.5- to- 8.4^36^). The water recovered after the cycles showed significant acidification, with a pH reduction of approximately 1 unit. This acidification may result from adsorption and accumulation of basic salts by the treated specimens. Reduction of pH in the surrounding medium may benefit the stability of calcium alginate, as its typical pKa is around 3.5, making it more chemically stable in solutions with lower pH^37^.

#### Micro-scale analysis

**Fig. 6:**
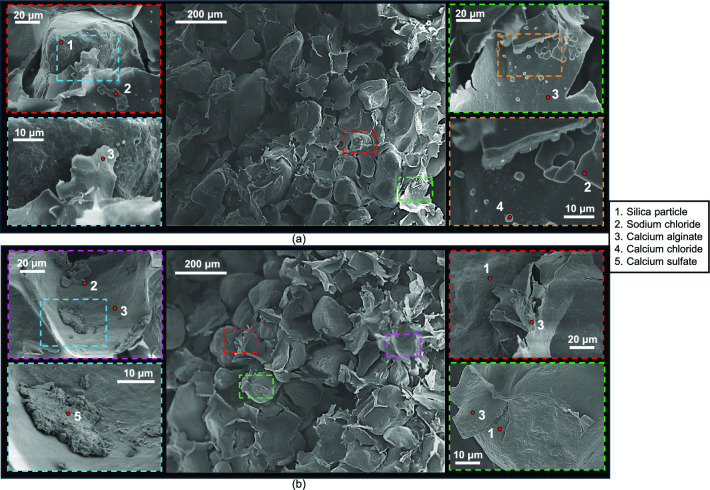
Micro-scale characterisation of treated sand with 4.6% sodium alginate after 7 wet-dry cycles of seawater.

**Fig. 7:**
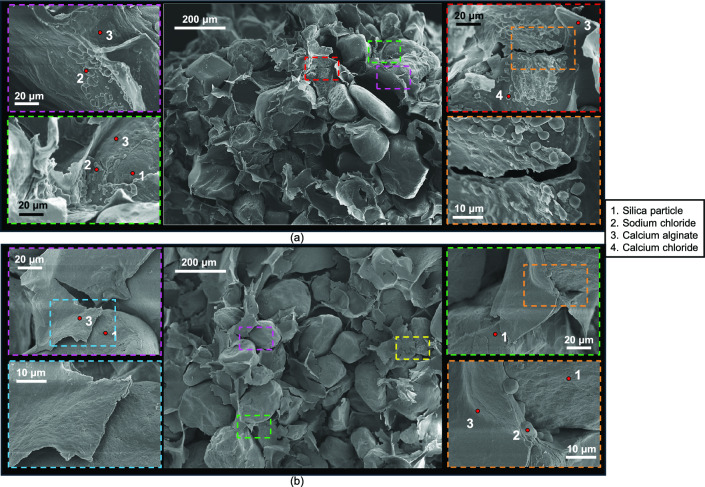
Micro-scale characterisation of treated sand with 4.6% sodium alginate after 14 wet-dry cycles of seawater.

**Fig. 8:**
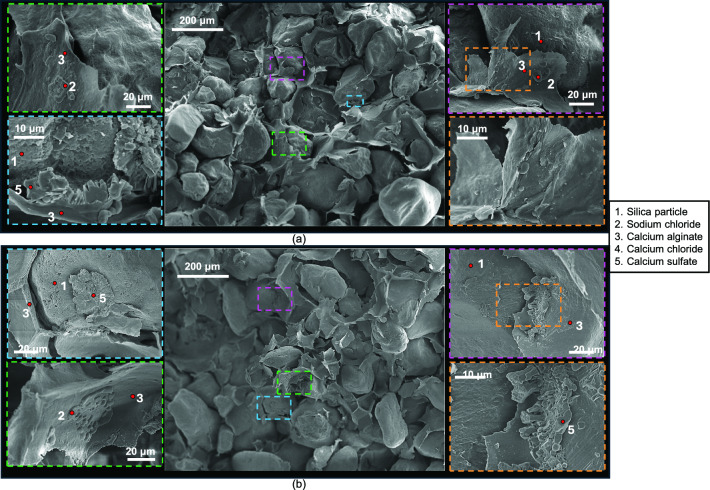
Micro-scale characterisation of treated sand with 4.6% sodium alginate after 28 wet-dry cycles of seawater.

Figures 6, 7 and 8 show SEM images with EDS-based labels of 4.6% alginate specimens after 7, 14 and 28 wet-dry cycles. Calcium alginate membrane-like structure consistently wrapped silica sand particles, with no evidence of reversion to sodium alginate. Numerous tears were observed in the membranes, although many remained partially adhered to sand particles (Fig. 6b lower-right image, Fig. 7b top left image). Accumulations of precipitated sodium chloride, calcium chloride and calcium sulphate crystals became increasingly apparent on the alginate surfaces and at the membrane-particle interfaces, with greater quantities observed after 28 cycles.

## Analysis and discussion

### Maximum strength: optimal sodium alginate content and mixing strategy

In general, when characterising soil properties, the goal is to obtain homogeneous samples, as weaker heterogeneities tend to govern overall soil strength^38^. Therefore, identifying the maximum strength achievable within acceptable deformation limits using crosslinked sodium alginate required investigating both the optimal sodium alginate content and the most suitable mixing strategy.

Two mixing strategies were used in this study: (1) wet mixing for concentrations between 1.4 and 2.3%, and (2) dry-mixing for concentrations between 2.3 and 10%. Wet mixing ensured full hydration of the alginate powder before crosslinking; however, it produced increased heterogeneity at higher alginate concentrations (≥ 2.3%) due to mixing difficulties. In contrast, dry mixing enabled more uniform distribution of alginate powder throughout the soil volume but may have increased heterogeneity in powder hydration.

The formation of calcium alginate membranes around silica sand particles produced a significant increase in UCS compared with untreated specimens (Fig. 4, dry untreated clean sand without cementation theoretically has a UCS = 0 MPa). Nevertheless, specimens prepared with sodium alginate concentrations between 8 and 10% underwent significant deformation during the crosslinking stage, resulting in a pronounced reduction in strength. This deformation likely arose from incomplete hydration of alginate powder during dry mixing: anhydrous alginate trapped at sand-particle contacts within the specimen hydrated belatedly during immersion in the calcium chloride solution, leading to excessive expansion (see pictures in Fig. 4). Based on the observed strengths and structural integrity, a sodium alginate concentration of 4.6% is suggested as optimal.

Both dry and wet mixing methods resulted in similar strengths in specimens prepared with 2.3% alginate. At this alginate content (upper limit for alginate dissolution in this study), the pore fluid volume was sufficient to fully hydrate the biopolymer powder. This finding highlights the importance of thorough mixing during sample preparation when using biopolymers, regardless of the mixing strategy. It also underscores the need to ensure sufficient pore fluid is available for powder hydration, either during sample preparation or crosslinking (the latter potentially leading to deformation, as observed in this study). Given the practicality of the dry mixing approach, particularly at lower alginate concentrations, it is recommended for future applications.

### Resilience of alginate-treated sands to seawater wet-dry cycles

Based on sodium alginate composition (Eq. 1), and the total alginate dry weight on each sample subjected to wet-dry cycles, we can anticipate the potential ion exchange as the maximum quantity of calcium/sodium that each sample can hold/release (Table 2). During the crosslinking stage, we exposed each sample to 1.56 g of dissolved calcium, which is sufficient to replace sodium in alginate chains on each case. This analysis indicates that before wet-dry cycles, each specimen has its maximum calcium concentration within the alginate bonds (Table 2).

**Table 2:** Anticipated sodium/calcium content depending on alginate concentration.

Alginate content [%]	Alginate dry weight [g]	Maximum Na content [g]	Maximum Ca content [g]
1.4	1.2	0.14	0.12
2.3	2	0.23	0.21
4.6	3.98	0.46	0.41

Based on the artificial seawater composition and the volumes used (see methodology section for details), each specimen had 2.26 g of sodium available during each wetting event, a much higher quantity than the maximum quantities that it could hold in each case (Table 2). High concentrations of sodium in the fluid were aimed to drive the replacement of calcium ions by sodium in biopolymer chains. Consequently, it was anticipated that significant calcium release (up to 0.41 g) and sodium uptake (up to 0.46 g) would occur during wet-dry cycling.

However, fluid analyses revealed no significant change in calcium concentration in all the fluids tested and an increase in sodium from the 12th cycle (Fig. 5b and c). These findings, together with SEM–EDS characterisation (Figs. 6, 7 and 8), indicate the chemical stability of the calcium alginate membranes, even under repeated exposed to high concentrations of dissolved sodium.

Despite this chemical stability, treated sands experienced significant reductions in compressive strength over repeated cycles. Mechanical damage on the treated specimens was driven by two phenomena:

1. Expansion–contraction-induced membrane degradation. Repeated wetting and drying subjected calcium alginate membranes to cyclical expansion and contraction, likely leading to tearing and weakening, as evidenced by SEM images showing partially detached membranes. A similar degradation process could have taken place in the study by Wen et al. (2019)^16^, where no salinity was involved, yet wet-dry cycles resulted in decrease in strength.
2. Crystallisation pressure tearing. Accumulation and precipitation of sodium chloride, calcium chloride, and calcium sulphate crystals were observed at the membrane–particle interface. In this case, wetting fluid permeates to the particle-membrane interface; then, water molecules evaporate during drying resulting in salt re-precipitation. Crystallisation pressures for the above-mentioned crystals range from 1 to over 50 MPa^39–41^, resulting in enhanced damage and detachment of the membranes.

Both damage mechanisms are associated with the high permeability of treated specimens, which allows seawater to readily infiltrate the pore network, promoting membrane weathering and salt precipitation. Therefore, future studies should consider a combination of sodium alginate with microbial-driven precipitation, or fungal-driven hydrophobicity to further reduce the permeability of the specimens and enhance their durability. Other limitations of this study for its direct field applications include the use of quartzitic poorly graded sand, which, while ensuring repeatability and facilitating interpretation of degradation mechanisms, may not capture the full variability of natural soils. In addition, the effect of wave action should be addressed in future work to validate the findings for field applications.

## Conclusions

This study has presented an experimental investigation complemented by theoretical analyses to assess the use of sodium alginate as a soil-improvement method in coastal environments. Major findings are summarised below:

- Results demonstrate that sodium alginate effectively increases the compressive strength of clean sands by forming stable calcium alginate membranes around sand particles.
- Residual calcium alginate emerges as the highest strength producer in comparison to ultra-low viscosity and standard sodium alginates.
- An optimal alginate concentration of 4.6% was identified, resulting in the highest UCS and specimen integrity. The obtained UCS strength is significantly higher than that achieved in other studies in the literature.
- The choice of mixing technique (dry versus wet) exhibited negligible influence on strength at low concentrations, while dry mixing proved more practical.
- Durability tests revealed that although calcium alginate membranes remained chemically stable under repeated wet-dry cycling in artificial seawater—supported by consistent calcium levels and microstructural analyses—specimens experienced 26–37% reductions in UCS after 28 cycles.
- Mechanical degradation resulted primarily from expansion–contraction-induced tearing of the membranes and damage caused by crystallisation pressures from salt precipitates.

The findings indicate that while sodium alginate treatment shows potential as an environmentally friendly soil improvement method for coastal applications, additional measures such as combining alginate treatment with techniques to reduce specimen permeability (e.g., microbial-induced calcite precipitation or hydrophobic amendments) should be explored to improve long-term durability.

## Supplementary Information

Supplementary Information.

## Data Availability

All data, models, and code generated or used during the study appear in the submitted article.
