# Supplementary material for: Assessment of the use of sodium alginate for soil improvement in coastal applications
Source: Sci Rep. 2025 Nov 5;15:38714. doi: 10.1038/s41598-025-22427-y (PMC12589614; doi:10.1038/s41598-025-22427-y)
Supplement: Supplementary file 1 — Supplementary Information. [file 41598_2025_22427_MOESM1_ESM.pdf]

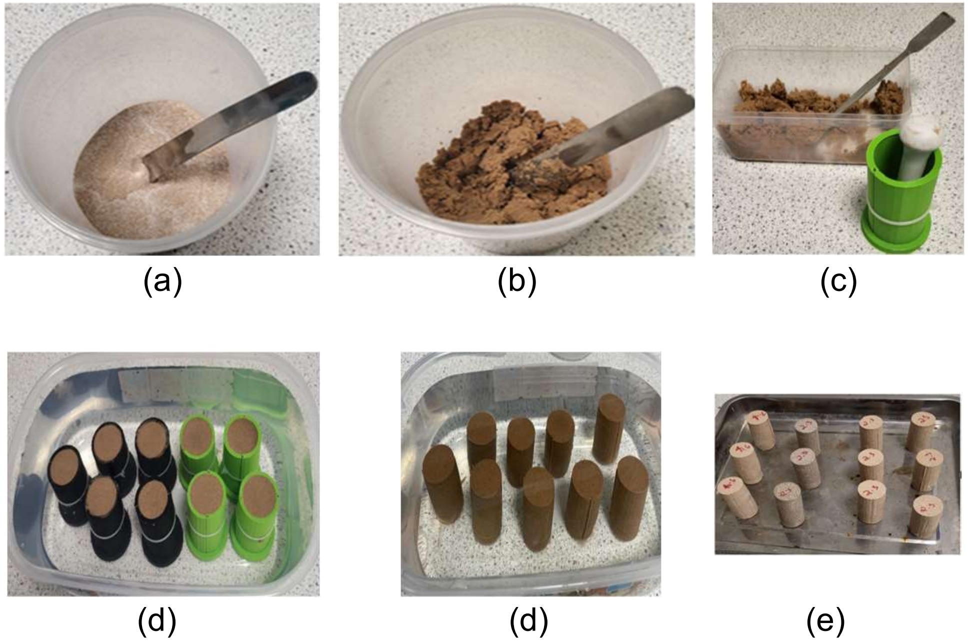


**S1.** Photographic images of the sample preparation procedure during (a) dry mixing, (b) wet mixing, (c) compaction, (d) crosslinking – 2 days, (e) crosslinking – 1 day, (f) curing
